# Mental Stress and Cognitive Deficits Management

**DOI:** 10.3390/brainsci14040316

**Published:** 2024-03-27

**Authors:** Fares Al-Shargie, Sahar Mohammed Taresh, Abdulhakim Al-Ezzi

**Affiliations:** 1 Electrical Engineering, American University of Sharjah, Sharjah P.O. Box 26666, United Arab Emirates; 2 Alfares for Research and Development Consultancy, Dubai 341041, United Arab Emirates; 3 Faculty of Social Science, Arts and Humanities, Lincoln University College, Petaling Jaya 47301, Malaysia; sahartaresh@yahoo.com; 4 Neurosciences, Huntington Medical Research Institutes, Pasadena, CA 91105, USA

Mental stress is a prevalent aspect of contemporary life that affects individuals from diverse backgrounds. Its correlation with cognitive impairment has attracted significant interest in the fields of psychology, neuroscience, and healthcare. Understanding the interplay between mental stress and cognitive deficits is crucial for developing effective management strategies to mitigate adverse outcomes. Extensive research has focused on the neurobiological processes that underlie the relationship between mental stress and cognitive decline [1]. It has been established that chronic stress leads to changes in brain structures and function, particularly the hippocampus, amygdala, and prefrontal cortex, which are critical for memory, learning, and executive functions [2,3,4]. Alterations in neurotransmitters, such as cortisol and catecholamines, play a significant role in stress-related cognitive impairments.

Stress management techniques, such as cognitive behavioral therapy (CBT) and stress management programs, have been shown to effectively mitigate mental stress and enhance mental health [5,6]. In particular, CBT is effective in reducing stress-related disorders and improving mental health in both clinical and general populations [5]. Additionally, stress management programs that utilize a cognitive behavioral approach have been shown to significantly improve mental health in the mothers of children with attention deficit hyperactivity disorder [7]. Such programs reduced stress levels and improved mental health outcomes, such as anxiety and depression, in these mothers. Therefore, these findings suggest that stress management interventions, such as CBT and cognitive behavioral stress management programs, can be effective in managing mental stress and improving cognitive deficits.

In addition, studies have found that mental stress impacts specific cognitive areas, such as attention, working memory, and decision making. Workplace stress and academic pressure contribute significantly to cognitive deficits, emphasizing the need for tailored interventions in these environments. The consequences of this stress are observable in the form of various behavioral symptoms, including a decreased ability to adapt and reduced speed in processing information. It is crucial to recognize these shortcomings to create tailored interventions that promote better cognitive performance when under stress. Despite the potential for lasting impacts on well-being, not all individuals who experience stress will develop adverse outcomes. Consequently, researchers frequently explore resilience scores to understand the factors that contribute to an individual’s ability to withstand and adapt to stress without succumbing to detrimental psychological effects [8]. A variety of interventions have been explored for managing cognitive deficits related to mental stress, including cognitive behavioral therapies, mindfulness-based approaches, and pharmacological interventions [9,10,11]. Although these interventions have shown promise in improving stress-induced cognitive impairments, their efficacy, limitations, and long-term effects require examination.

Several papers have contributed to this Special Issue aimed at gathering behavioral and neuroimaging studies on the assessment and mitigation of mental stress and cognitive deficiency. In [12], a method is proposed to identify the brain regions that are the most sensitive to detecting mental stress states via electroencephalography (EEG), with the potential to develop accurate wearable technologies for the real-time diagnosis of mental stress. This study emphasizes the role of optimal EEG channel selection in stress recognition, contributing to efficient and accurate stress monitoring using neuroimaging modalities. Likewise, ref. [13] examines the relationship between EEG indicators (particularly parietal and frontal alpha activity) and tinnitus severity, as well as the impact of background noise levels on these parameters. The findings suggest that tinnitus influences listening effort, highlighting the complex interplay between auditory processing, cognitive load, and stress in individuals with this condition. In particular, this study provides preliminary insights into the neurophysiological correlates of listening effort in tinnitus patients and suggests a potential link between listening effort, stress, and cognitive impairment. Meanwhile, ref. [14] examines the psychometric properties of two Portuguese versions of the Geriatric Depression Scale (GDS-27 and GDS-15) in a sample of Portuguese older adults with mild-to-moderate cognitive impairment. This study analyses the internal consistency, reliability, and construct validity of GDS-27 and GDS-15. The findings suggest that both GDS-27 and GDS-15 are reliable and valid instruments for assessing depressive symptoms in Portuguese-speaking older adults with cognitive impairment. Another study in [15] provides valuable insights into the cognitive complaints experienced by individuals during the recovery phase from acute COVID-19. The study’s findings have important implications for detecting, evaluating, and treating cognitive complaints in clinical practice. The study contributes to the growing body of knowledge on the cognitive effects of COVID-19 and provides valuable information for healthcare professionals and researchers involved in the care and study of individuals with post-acute sequelae of COVID-19. Finally, the study in [16] highlights the detrimental effects of Depressive Emotion (DE) on spatial cognition and provide evidence of brain resource reorganization to compensate for cognitive decline. The results emphasize the importance of understanding the neural mechanisms underlying DE’s interference with spatial cognition, shedding light on potential avenues for cognitive interventions in individuals with DE. The study’s comprehensive approach, combining EEG network analysis with behavioral observations, contributes to a deeper understanding of the cognitive impairments associated with DE. These findings have implications for the development of targeted interventions to address spatial cognition deficits in individuals with DE. Overall, this Special Issue contains five studies; more studies that combine multi-sensory stimulation are required.

Technologies, such as neurofeedback, virtual reality, transcranial magnetic/electric stimulation, auditory stimulation, and mobile applications, should be integrated as tools for managing mental stress associated with cognitive deficits [17,18,19,20]. These technologies have the advantage of integration into traditional therapeutic approaches to enhance accessibility and effectiveness in stress management. Furthermore, significant advancements have been made regarding unisensory stimulation, but a number of questions still remain unanswered. For example, it is not known whether stress management prevents stress-related disorders. To improve this situation, future studies should delve deeper into the intricacies of how stress affects people differently, refine intervention methods, and investigate the long-term impact of stress management on cognitive outcomes. Furthermore, it is crucial to examine the relationship between mental stress and other mental health issues.

Research should not only establish consistent and relevant measures of stress but also elucidate the connection between improved stress management or reduced stress levels and a decrease in the risk of well-established stress-associated disorders, including depression, anxiety, cardiovascular disease (CVD), and mild cognitive impairment. It is important for future studies to investigate the long-term impact of stress mitigation at various stages of recovery. This would aid in determining whether individuals with cognitive deficits who receive preventive measures early or late in the recovery process are less likely to develop stress-related problems over time.

An innovative approach to enhance the management of mental stress and cognitive deficits may involve integrating multiple interventions while harnessing the capabilities of the Internet of Things (IoT) and artificial intelligence (AI). By incorporating wearable devices into this comprehensive strategy, individuals and healthcare professionals can gain real-time insights into the state of mental health, facilitating timely interventions to prevent and mitigate potential negative consequences. To propel this field forward, future research should focus on a spectrum of pioneering approaches. The development of individualized stress profiles could pave the way for bespoke intervention strategies, utilizing advanced diagnostics to tailor treatments to individual stress responses and cognitive effects. Moreover, longitudinal studies ought to be conducted in order to map the trajectories of mental stress and its cognitive repercussions over extended periods.
